# Response of total phenols, flavonoids, minerals, and amino acids of four edible fern species to four shading treatments

**DOI:** 10.7717/peerj.8354

**Published:** 2020-01-13

**Authors:** Yanlin Wang, Shanshan Gao, Xingyuan He, Yan Li, Yue Zhang, Wei Chen

**Affiliations:** 1 Institute of Applied Ecology, Chinese Academy of Sciences, Shenyang, Liaoning, China; 2 University of Chinese Academy of Sciences, Beijing, China; 3 Key Laboratory of Forest Ecology and Management, Chinese Academy of Sciences, Shenyang, China; 4 Shenyang Arboretum, Chinese Academy of Sciences, Shenyang, China

**Keywords:** Shading, Edible fern species, Total phenols, Flavonoids, Amino acids, Minerals

## Abstract

Total phenols, flavonoids, minerals and amino acids content were investigated in leaves of four fern species grown under four shading treatments with different sunlight transmittance in 35% full sunlight (FS), 13% FS, 8% FS and 4% FS. The leaves of four fern species contain high levels of total phenols and flavonoids, abundant minerals and amino acids, and these all were strongly affected by transmittance. Total phenols and flavonoids content were significantly positively correlated with transmittance, while minerals and total amino acids content were significantly negatively correlated with transmittance, a finding that supports research into how higher light intensity can stimulate the synthesis of phenols and flavonoids, and proper shading can stimulate the accumulation of minerals and amino acids. *Matteuccia struthiopteris* (L.) Todaro (MS) had the highest total phenols content, *Athyrium multidentatum* (Doll.) Ching (AM) showed the highest total amino acids, total essential amino acids content, *Osmunda cinnamomea* (L) var. *asiatica* Fernald (OCA) exhibited the highest total non-essential amino acids and flavonoids content.* Pteridium aquilinum* (L.) Kuhn var. *latiusculum* (Desy.) Underw. ex Heller (PAL) exhibited the highest minerals content. This research can provide a scientific basis for the cultivation and management of those four fern species.

## Introduction

China’s approximately 2,300 fern species (out of the global total of 12,000 fern species) are distributed in various environments, among which 300 species can be used as traditional Chinese medicine with a few of them popularly consumed as wild vegetables (Xiao, 2017; Zhang et al., 2012). Fern species are widely distributed in the Northeastern China, and up to eight fern species can be eaten, *Matteuccia struthiopteris* (L.) Todaro (MS), *Athyrium multidentatum* (Doll.) Ching (AM), *Osmunda cinnamomea* (L) var. *asiatica* Fernald (OCA) and *Pteridium aquilinum* (L.) Kuhn var. *latiusculum* (Desy.) Underw. ex Heller (PAL) are well known and rich in nutrients (Liu & Li, 1995; Liu & Wang, 2018).

Previous studies reported that the four fern species are rich in nutrients including antioxidants, minerals, amino acids, vitamins, etc (Dong et al., 1993; Liu, Yu & Wang, 2011; Qi et al., 2015; Yao, 2003; Yao et al., 2003; Zhao, Fu & Liu, 1991). MS and AM have multiple pharmacological effects such as heat-clearing, detoxifying, regulation of blood pressure and pain relief (Han et al., 2018; Zhu et al., 2016). Secondary metabolites extracted from OCA showed high antioxidant activity and a broad antibacterial spectrum (Qi et al., 2015). PAL also has some bioactivities like anti-viral and antibacterial properties (Wang & Wu, 2013).

Phenols and flavonoids are common secondary metabolites in plants, which not only regulate the growth and development (Agati & Tattini, 2010; Manoj & Murugan, 2012), but also have important health benefits for human (Andrae-Marobela et al., 2013; Chen et al., 2018). Phenols’ roles within plants include being part of their defense against herbivores, weeds and pathogens, and phenolic compounds serve as structural support in plants (Jones & Hartley, 1999; Otálora et al., 2018). Phenolic compounds have been found to be important for the sensory, nutritional, medicinal and commercial value of edible and medicinal plants (Otálora et al., 2018; Wahle et al., 2010). Flavonoid compounds have important physiological and ecological functions for plants, for example in protecting them from UV radiation by scavenging reactive oxygen species (ROS) due to their cytotoxicity and ability to interact with enzymes (Heim, Tagliaferro & Bobilya, 2002; Treutter, 2006; Vaknin et al., 2005). Flavonoids in foods contribute to human health by assisting in preventing diseases associated with oxidative stress (Pourcel et al., 2007; Williams, Spencer & Rice-Evans, 2004).

For plants and human growth, minerals are essential (Hänsch & Mendel, 2009; Mir-Marqués, Cervera & De la Guardia, 2016), with some involved in photosynthesis, mitochondrial repair, carbon and nitrogen metabolism, and active oxygen scavenging. Some mineral elements can be used in human body as a component of proteins and enzymes (Hänsch & Mendel, 2009; Maathuis, 2009; Mir-Marqués, Cervera & De la Guardia, 2016). Lack of mineral elements in the human diet has been found to cause serious problems, especially for children and pregnant women (Paiva et al., 2017b). Amino acids involve a range of physiological activities in plants and in the human body, they are used to synthesize proteins, maintain nitrogen balance, and produce antibodies and certain hormones in humans (Hildebrandt Tatjana et al., 2015; Sonawala et al., 2018; Zhao et al., 2018).

Light’s influence on plant growth is well-known, and its intensity has an obvious impact on plant growth and physiology (Chen et al., 2017; Shao et al., 2014). But, more specifically, light intensity affects the accumulation of some secondary metabolites and nutrition. Light with higher intensity is known to stimulate the synthesis of phenols and flavonoids to protect the living plants (Liu et al., 2018; Riachi et al., 2018), and studies have shown that light intensity can affect the accumulation of amino acids and minerals (Riga et al., 2019; Stagnari, Galieni & Pisante, 2015; Zrig et al., 2016). However, the effects of light intensity on the secondary metabolites and nutrient accumulation of edible fern species have not been reported. In this study, total phenols, flavonoids, minerals and amino acids content were investigated using the leaves of four edible fern species (MS, AM, OCA, and PAL), plants were grown under four shading treatments with different transmittance of 35% full sunlight (FS), 13% FS, 8% FS and 4% FS to examine the effects of light intensity on the secondary metabolites and nutrient content of the four edible fern species.

## Materials & Methods

### Experimental site

The study was conducted in an open and unshaded area of the Arboretum of the Institute of Applied Ecology, Chinese Academy of Sciences (41°46′N, 123°27′E), which has a mean elevation of 45 m above sea level.

### Plant materials and experiment design

Four fern species commonly found in Northeast China, namely *Matteuccia struthiopteris* (L.) Todaro (MS), *Athyrium multidentatum* (Doll.) ching (AM), *Osmunda cinnamomea* (L.) var. *asiatica* Fernald (OCA) and *Pteridium aquilinum* (L.) Kuhn var. *latiusculum* (Desy.) Underw. ex Heller (PAL), were used in an outdoor pot experiment. The rhizomes of four fern species were collected on April 25, 2018, a day when the average daily temperature was 15 °C and the relative humidity of the air was 52%. Three-year-old rhizomes of the four fern species were transferred to pots (caliber 21 cm × depth 14 cm), which were filled with turfy soil and sand mixed at a volume ratio of 3:1 (v/v). The organic matter content was 52%, and the N:P:K was 23:4:8, the plants were fully watered for cultivation from April 25, 2018 to May 15, 2018. Climatic conditions during the cultivation period are shown in Fig. S1. After plants’ growth traits were stable, the plants with consistent height and good growth were used for experimental studies on May 15, 2018. The experiment consisted of four treatments. Each treatment used all four fern species and made three repetitions. Fern plants were placed under four black shading nets of different specifications. Based on the light conditions for the growth of fern species in the wild, different transmittances were chosen for simulation. After measuring the light intensity under full sunlight and shade, the light transmittances of the four shading nets were determined to be 35% full sunlight (35% FS), 13% full sunlight (13% FS), 8% full sunlight (8% FS) and 4% full sunlight (4% FS). After 60 days of shading, the relevant indicators were determined. Climatic conditions during the treatment is showed in Fig. S2.

### Sample processing and preparation

The leaves of four fern species under four shading treatments were collected respectively, rinsed with running water first and distilled water afterwards. The washed leaves then were placed in clean envelopes and dried for 30 min at 105 °C in an electric blast drying oven (Zhongxing, China). They were dried at 60 °C to a constant weight, and the dried leaves were ground with a grinder to make possible the detection and analysis of total phenols, flavonoids, mineral elements and amino acids content.

### Determination of total phenols content

The dried leaves powder’s total phenols content were tested by means of a total phenols test kit (Solarbio, Beijing, China). Under alkaline conditions, the phenolic substance reduced the tungsten molybdic acid to produce a somewhat blue compound with a characteristic absorption peak at 760 nm. Absorbance at 760 nm was measured to obtain the sample’s total phenols content. About 0.1 g of dried leaves powder was weighed, 2.5 mL of extract solution was added, and total phenols were extracted by ultrasonic extraction. The ultrasonic power was 300 W, the mixture was broken for 5s, intermittent 8s, and then extracted for 30 min at 60 °C. The mixture was centrifuged for 10 min at 12,000 rpm, at 25 °C, and the supernatant was taken and diluted to 2.5 mL with extract solution. The absorbance of the extraction was measured at wavelength of 760 nm using a Microplate reader (InterMed, South Portland, ME, USA). The standard curve was tested with 1 mg mL^−1^ tannic acid standard solution.

### Determination of flavonoids content

The flavonoids content in the fern leaf powder was tested using a flavonoid test kit (Solarbio, Beijing, China). In alkaline nitrite solution, flavonoids and aluminum ions form a red complex with a characteristic absorption peak at 470 nm. Absorbance of the sample extract at 470 nm was used to calculate the sample’s flavonoids content. About 0.1 g of dried leaves powder was weighed, 1.0 mL of extract solution was added, and total phenols were extracted by ultrasonic extraction. The ultrasonic power was 300 W, the mixture was broken for 5s, intermittent 8s, and then extracted for 30 min at 60 °C. The mixture was centrifuged for 10 min at 12,000 rpm, at 25 °C, the supernatant was taken and diluted to 1.0 mL with the extract solution. Absorbance of the extraction was measured at wavelength of 470 nm using a Microplate reader (InterMed, South Portland, ME, USA). The standard curve was tested with 10 mg mL^−1^ tannic acid standard solution.

### Determination of minerals content

Approximately 0.5 g of dried leaves powder was analyzed for the content of K, Ca, Mg, Fe, Mn, Cu, Zn, Na. The mineral elements were extracted by nitric-perchloric acid digestion (Bystriakova, Bader & Coomes, 2011). The leaf samples were placed into clean beakers, and then 20 mL of nitric acid (65%) with 5 mL of perchloric acid (70%) was added, the mixture was stayed overnight. After nitric-perchloric acid digestion, 2% nitric acid was added into the beaker and diluted to 25 mL. Using an ICP-OES (Agilent, America), the absorbance of solution was measured at wavelength 766.5 nm (K), 317.9 nm (Ca), 279.6 nm (Mg), 238.2 nm (Fe), 257.6 nm (Mn), 327.4 nm (Cu), 213.9 nm (Zn), 589.6 nm (Na). The standard solutions (1,000 µg mL^−1^) used for calibration were purchased from Tianjin Guangfu Fine Chemical Research Institute (China).

### Determination of amino acids content

The fern’s amino acids content was measured according to Yang et al. (2002). Approximately 1.0 g dried leaves powder was placed into digestion bottle, 20 mL of 6 mol L^−1^ HCl was added, and the mixture was digested for 24 h at 110 °C. After digestion, the mixture was filtered and diluted to 100 mL with ultrapure water. The solution obtained in the previous step (2 mL) was added into a beaker and evaporated in a 60 °C water bath, after which the crystallization was dissolved with 0.02 mol L^−1^ HCl and filtered to a volume of 2 mL. This mixture was measured for the amino acids content by using an amino acids analyzer (Hitachi Japan).

### Statistical analysis

All data were analyzed with Microsoft Excel 2016 and SPSS 22.0 software. Graphs were edited with GraphPad Prism 5.0 software. Analysis of variance (ANOVA) and correlation analysis (Pearson) were performed using SPSS 22.0 software, and means were compared based on Duncan’s test at *P* ≤ 0.05.

## Results

### Total phenols and total flavonoids content

**Figure 1:**
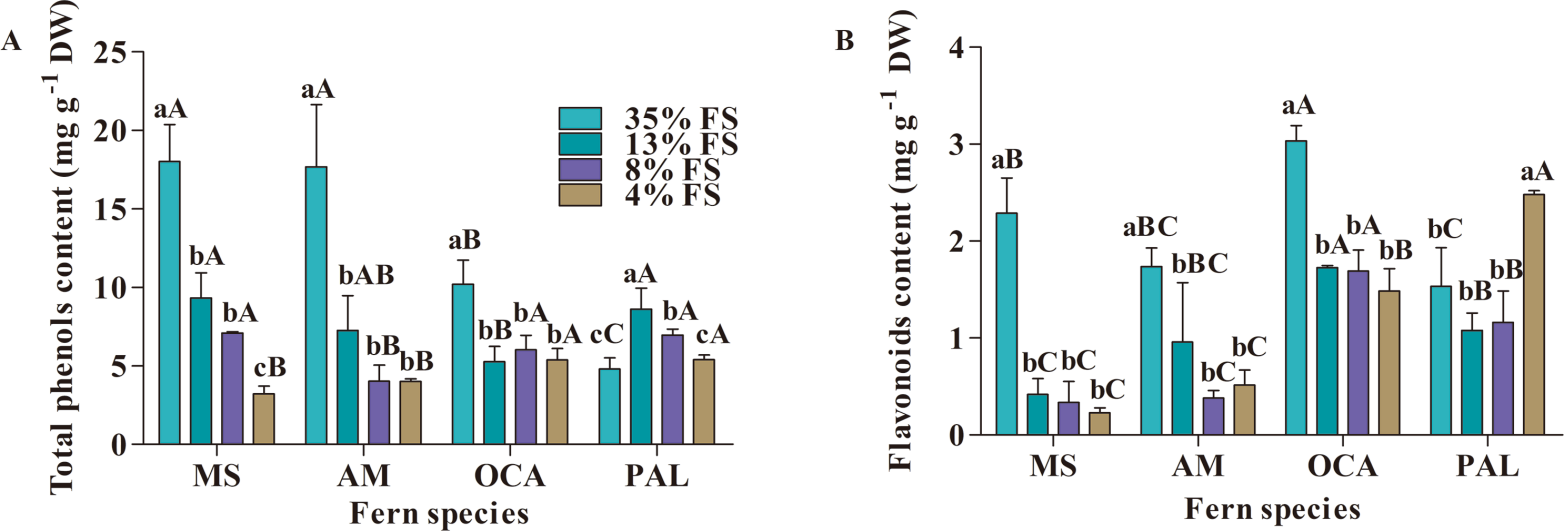
Effect of shading on total phenols and flavonoids content (mg g^−1^ DW) in four fern species. (A) Total phenols content. (B) Flavonoids content. MS, M. struthiopteris, AM, *A. multidentatum*, OCA = *O. cinnamomea* (L.) var. *asiatica*, PAL = *P. aquilinum* (L.) Kuhn var. *latiusculum*, 35% FS, 35% full sunlight, 13% FS, 13% full sunlight, 8% FS , 8% full sunlight, 4% FS= 4% Full sunlight. Different lowercase letters mean significant difference among different treatments in the same fern at *P* ≤ 0.05. Different uppercase letters mean significant difference between four fern species at *P* ≤ 0.05 (Duncan’s test). Error bars are ± SD (*n* = 3).

The study found that total phenols and flavonoids content in leaves of four fern species were significantly affected by transmittance (Figs. 1A and 1B). MS, AM and OCA exhibited the highest total phenols and flavonoids content in 35% FS, but PAL showed the highest total phenols in 13% FS and flavonoids content in 4% FS. For MS, the lowest total phenols and flavonoids content appeared in 4% FS. The lowest total phenols and flavonoids content for AM appeared in 4% FS, and for OCA it was in 8% FS. PAL showed the lowest total phenols content in 35% FS and lowest flavonoids content in 13% FS. However, the flavonoids content of PAL increased under the treatment of 13% FS, 8% FS and 4% FS.

Among the four fern species, total phenols and flavonoids content largely differed from fern species to species. For example, the content of total phenols of MS was higher than that of the three other fern species, while the flavonoids content of MS was lower than that of the other three.

### Minerals content

**Figure 2:**
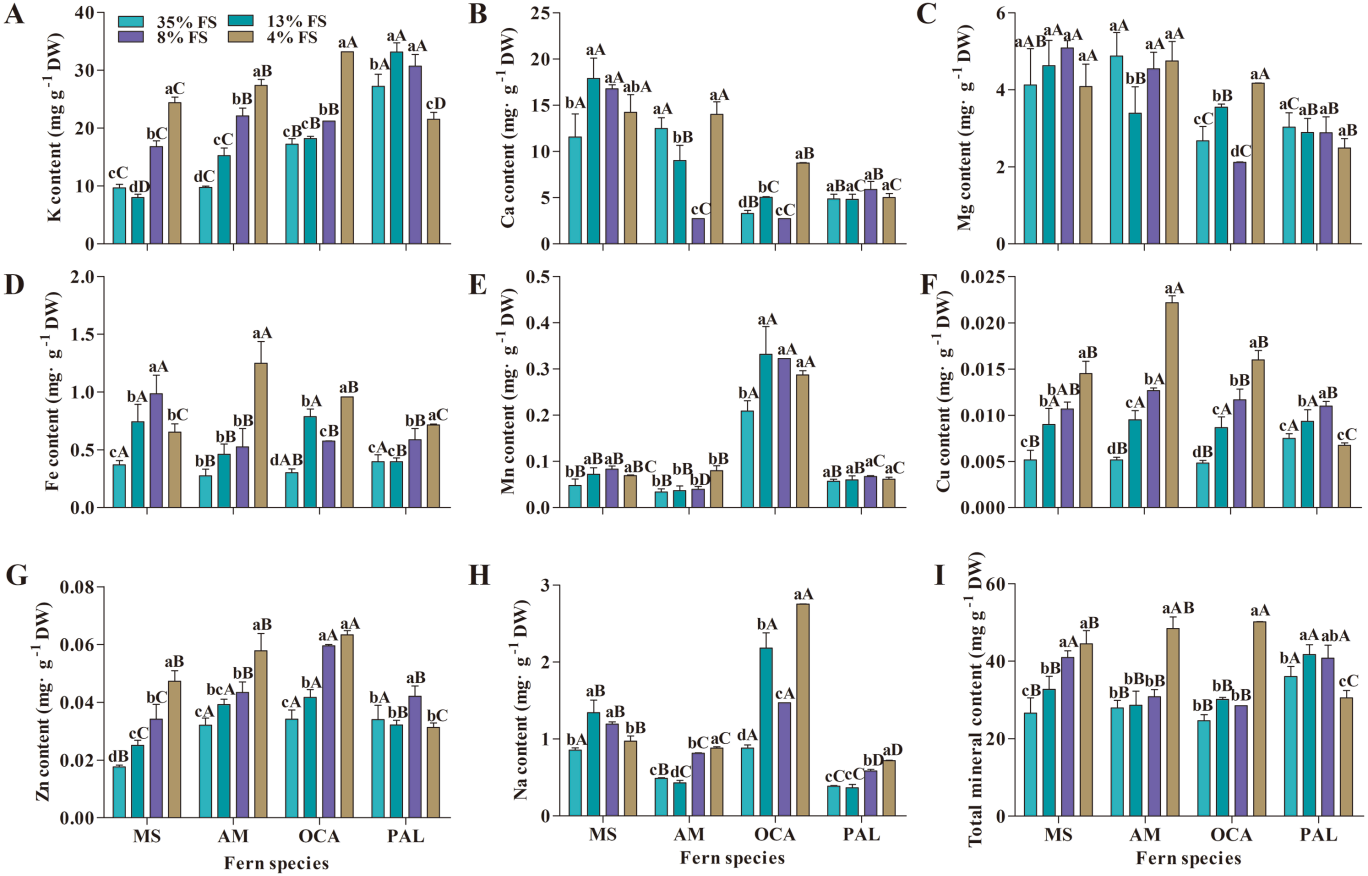
Effect of shading on minerals content (mg g^−1^ DW) in four fern species. (A) K content; (B) Ca content; (C) Mg content; (D) Fe content; (E) Mn content; (F) Cu content; (G) Zn content; (H) Na content; (I) Total mineral content. MS, *M. struthiopteris*, AM, *A. multidentatum*, OCA, *O. cinnamomea* (L.) var. *asiatica*, PAL, *P. aquilinum* (L.) Kuhn var. *latiusculum*, 35% FS, 35% full sunlight, 13% FS, 13% full sunlight, 8% FS, 8% full sunlight, 4% FS, 4% full sunlight. Different lowercase letters mean significant difference among different treatments in the same fern at *P* ≤ 0.05. Different uppercase letters mean significant difference between four fern species at *P* ≤ 0.05 (Duncan’s test). Error bars are ± SD (*n* = 3).

The minerals content in leaves of the four fern species was significantly affected by transmittance (Fig. 2). In this study, the highest content of a majority of minerals of MS, AM and OCA appeared in lower transmittance (8% and 4% FS). Conversely, the lowest content of most mineral elements appeared at 35% FS. The K, Cu, Zn content of MS, AM and OCA expressed an upward trend in 13% FS, 8% FS and 4% FS. The K, Cu, Zn content in 4% FS were significantly higher than 13% FS and 8% FS, except the Zn content of OCA.

The change of PAL in the minerals content was the most complicated finding. PAL exhibited the highest K and Mg content in 13% FS and 35% FS, the highest Ca, Fe, Na content in 4% FS, the highest Mn, Cu and Zn content in 8% FS. The lowest K, Mg, Cu and Zn content was recorded in 4% FS, the lowest Ca in 8% FS, the lowest Na content in 13% FS, and the lowest Fe and Mn content was recorded in 35% FS. For MS, AM and OCA, the highest total minerals content appeared in 4% FS, and the lowest total minerals content was recorded in 35% FS, whereas PAL exhibited the highest total minerals content in 13% FS, and the lowest total minerals content in 4% FS.

The content of each mineral element varies greatly among the four fern species. The highest K and total minerals content were observed in PAL; the highest Ca and Fe content were recorded in MS; AM had the highest Mg and Cu content, while OCA had the highest Mn, Zn and Na content.

### Amino acids content

**Figure 3:**
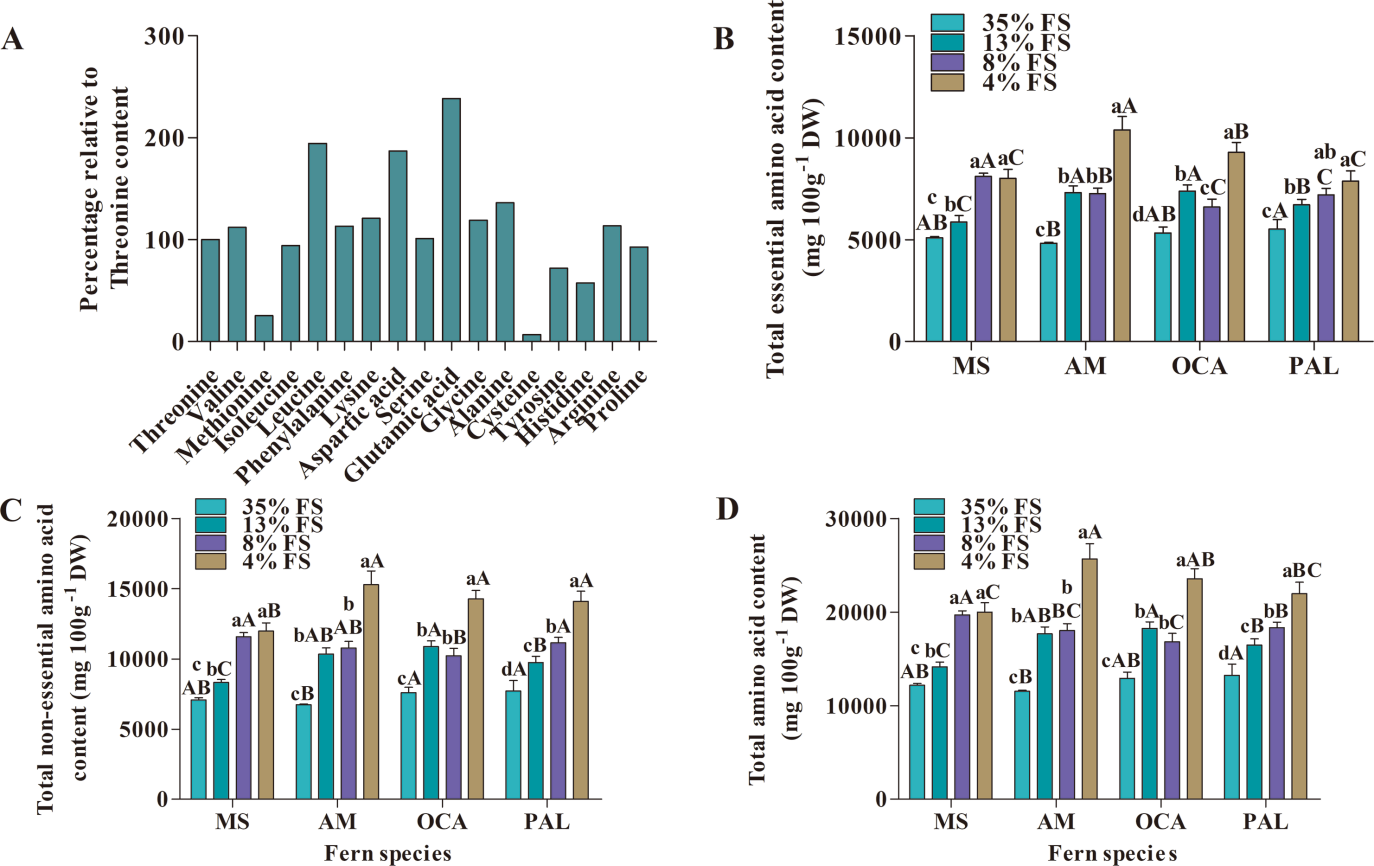
Changes of amino acids content (mg 100 g^−1^ DW) of four fern species under four shading treatments. (A) Relative value of each amino acid change (percentage relative to Threonine). (B) Essential amino acid content. (C) Non-essential amino acid content. (D) Total amino acid content. MS, *M. struthiopteris*, AM, *A. multidentatum*, OCA, *O. cinnamomea* (L.) var. *asiatica*, PAL, *P. aquilinum* (L.) Kuhn var. *latiusculum*, 35% FS, 35% full sunlight, 13% FS, 13% full sunlight, 8% FS, 8% full sunlight, 4% FS, 4% full sunlight. Different lowercase letters mean significant difference among different treatments in the same fern at *P* ≤ 0.05. Different uppercase letters mean significant difference between four fern species at *P* ≤ 0.05 (Duncan’s test). Error bars are ± SD (*n* = 3).

The amino acids content in leaves of four fern species also was significantly affected by four shading treatments with different transmittance. Altogether, 16 amino acids were detected, including 7 essential amino acids and 9 non-essential amino acids (Fig. 3A). Among the essential amino acids, the leucine content was the highest and the methionine content was the least; for the non-essential amino acids, the glutamic acid content was the highest and the cysteine content was the least.

In addition to cysteine, the amino acids content in leaves of the four fern species increased with decreasing transmittance. The highest cysteine content of four fern species was observed in 8% FS; but MS, AM and PAL had the lowest cysteine content in 4% FS, while OCA had the lowest cysteine content in 35% FS (Figs. S3 and S4).

Except for cysteine, the content of most of the other amino acids in AM, OCA and PAL was significantly higher than other treatments under 4% FS; there was no significant difference in 13% FS and 8% FS. In addition, the amino acids content of MS was not significantly different between 8% FS and 4% FS, and was significantly higher than 35% FS and 13% FS, with the exceptions of aspartic acid and proline.

The highest total amino acid, total essential amino acids and total non-essential amino acids content of MS were recorded in 8% FS, and similarly for AM, the highest total amino acid and total non-essential amino acid content appeared in 8% FS; but the highest total essential amino acid content of AM was recorded in 13% FS. OCA in 13% FS exhibited the highest level of total amino acid, total essential amino acid and total non-essential amino acid. PAL in 4% FS exhibited the highest total amino acid and total non-essential amino acid content, PAL’s highest total essential amino acid content was in 13% FS (Figs. 3B–3D).

Across the four fern species investigated, AM was lower than the other three fern species in terms of each single amino acid, total amino acids, total essential and total non-essential amino acids content. MS had the highest essential amino acids content (except for methionine) and the highest readings for aspartic acid, alanine, tyrosine, proline and total amino acid and total essential amino acids content. OCA had the highest glycine, cysteine and total non-essential amino acids content. PAL was the highest for glutamic acid, histidine and arginine (Fig. S4).

## Discussion

Environmental conditions affect plant growth, development and distribution, and the nutrients of plants are also affected by environmental factors (Siracusa & Ruberto, 2014; Tounekti & Khemira, 2015; Tounekti et al., 2010). As an important ecological factor, light affects the photosynthesis of plants, also has a bearing on the chemical composition of plants (Fukuda, 2019; Kyriacou et al., 2016; Rouphael et al., 2018). Despite previous studies on the effects of light quality and light intensity on crop growth and quality (Fiutak et al., 2019; Frede, Schreiner & Baldermann, 2019; Kaiser et al., 2019; Ruangrak & Khummueng, 2019; Shibuya et al., 2019), few studies have involved under-forest economic crops such as wild vegetables. MS, AM, OCA and PAL are important wild vegetables in Northeast China (Liu & Li, 1995; Liu & Wang, 2018), but there was little research on the effects of light conditions on the quality of such four edible fern species. Understanding the effects of light conditions on the quality of those four edible fern species is critical to their cultivation, management and utilization for human consumption.

The relationship between light intensity and the synthesis of plant secondary metabolites is not known. Previous studies have shown that increased light can cause accumulation of flavonoids and total phenols in herbal medicines (Graham, 1998). Higher light can decrease the phenolic compounds in the roots of *Beta vulgaris* var. *conditiva* Alef. and in the leaves of lettuce plants (*Lactuca sativa L.*) (Galieni et al., 2015; Perez-Lopez et al., 2018; Stagnari et al., 2014). The shading treatments employed in this research significantly affected the total phenols and flavonoids content; the content of total phenols and flavonoids was significantly positively correlated with the transmittance of shading nets (*r* = 0.69 (*P* < 0.0001) and 0.52 (*P* = 0.0002), respectively) (Table S1), which indicates that light with high intensity contributes to the synthesis of phenols and flavonoids. The synthesis of total phenols by higher light intensity possibly is due to higher light-induced activation of phenylalanine amino lysis enzyme in the phenolic acid synthesis pathway (Kumari, Singh & Agrawal, 2009).

Though the synthesis of flavonoids induced by high light intensity has been found to have an association with the expression of phenylalanine ammonia lyase (Graham, 1998; Saito et al., 2013), the flavonoids content of PAL was on the rise in the last three treatments (13% FS, 8% FS and 4% FS), which is similar to the research of Shao (2010) and Song et al. (2017). This probably because biosynthesis of flavonoids in plant was affected by the light conditions in the environment, this change is also related to species (Shao, 2010; Song et al., 2017).

Light of high intensity can damage plant cells and produce ROS (Mullineaux et al., 2018; Pinto-Marijuan & Munne-Bosch, 2014; Szymańska et al., 2017), and plants can scavenge ROS by activating antioxidant systems (enzymatic antioxidant systems and non-enzymatic antioxidant systems). The non-enzymatic antioxidant systems include secondary metabolites such as ascorbic acid, carotenoids, and *α*-tocopherol (Georgieva et al., 2017; Kataria et al., 2019; Soares et al., 2018). Similarly, phenols and flavonoids can be used as ROS scavengers to remove ROS in plants (Franzoni et al., 2019; Liao, Greenspan & Pegg, 2019; Meini et al., 2019; Naikoo et al., 2019; Schenke et al., 2019; Xiang et al., 2019). In our study, across the shading treatment, the content of H_2_O_2_ (shown in raw data) was significantly positively with the content of total phenols and flavonoids content (*r* = 0.62 (*P* < 0.0001) and 0.41 (*P* = 0.0047), respectively) (Fig. S5), which indicates that the four fern species can scavenge H_2_O_2_ by increasing the synthesis of total phenols and flavonoids. As the light intensity increases, plants can alleviate the oxidative damage caused by strong light through the synthesis of secondary metabolites in the body (Agati & Tattini, 2010; Perez-Lopez et al., 2018).

Some studies have found that when *Salvinia* species are stressed by heavy metals, they can accumulate phenolic compounds against oxidative damage caused by heavy metals (Prado et al., 2016; Prado et al., 2013). Our previous study found that when AM, MS and *Adonis* species are subjected to drought stress, phenolic compounds can also be accumulated to alleviate the oxidative damage caused by drought (Gao et al., 2020; Wang et al., 2019). In the present study, total phenols and flavonoids increased with the increase of light intensity, and were significantly positively correlated with hydrogen peroxide content, indicating that secondary metabolites such as phenolic compounds are important for alleviating ferns’ oxidative damage.

Environmental factors also have an impact on the accumulation of mineral elements in plants (Paiva et al., 2017a; Sarker & Oba, 2018). Light intensity affects the accumulation of minerals in plants. Colonna et al. (2016) reported that ten leafy vegetables accumulated more K, Ca and Mg under low light intensity. Lettue under low light intensity exhibited higher K, Ca, Mg, Fe, Mn and Zn (Stagnari, Galieni & Pisante, 2015). The mineral content of the four fern species in this study showed similar change. The lowest mineral content was recorded in 35% FS, while K, Fe, Mn, Cu, Zn and Na content reached their maximum in 4% FS with rates, respectively, of 2.01, 2.82, 1.56, 2.83, 1.82 and 1.98 times that of 35% FS. The highest Ca and Mg content appeared in 8% FS. Among them, the content of K, Fe, Cu, Zn and Na were significantly negatively correlated with the transmittance of shading nets, and the correlation coefficients were −0.45 (*P* = 0.0012), −0.68 (*P* < 0.0001), −0.70 (*P* < 0.0001), −0.58 (*P* < 0.0001) and −0.36 (*P* = 0.0125) (Table S1). The total minerals content was negatively correlated with transmittance with correlation coefficients of −0.55 (*P* < 0.0001) (Table S1). The change of minerals indicates that moderate shading stimulates the accumulation of mineral elements, which is similar to the results of Colonna et al. (2016) and Lefsrud et al. (2006). This may be due to the increased light intensity that promotes photosynthesis of the plant, and the increased fresh weight of the plant causes dilution of the mineral element content (Jones, Wolf & Mills, 1996).

Previous studies reported that light can affect the free amino acids content (Riga et al., 2019; Zrig et al., 2016). The significant differences in free amino acids content between thyme plants (*Thymus vulgaris*) grown in different light conditions, the amino acids content in shading condition was higher than in open-field after four weeks (Zrig et al., 2016). Similarly, the free amino acids content of lettuce was affected by light intensity and global radiation (Riga et al., 2019). However, to the best of our knowledge, the effect of light intensity on plants’ total amino acids (protein amino acids and free amino acids) content of plants has not been reported. In our study, there were significantly differences in amino acids content among the four fern species grown under different shading nets. For most of the amino acids measured, their content increased with the decrease of transmittance of shading nets. In addition to cysteine content, the content of other single amino acid, total amino acids, total essential amino acids and total non-essential amino acids were all significantly negatively correlated with transmittance of shading nets (*P* < 0.0001) (Table S1), from which we can infer that lower light levels may contribute to amino acid accumulation in those four fern species. This may be due to a decrease in plants’ photosynthetic capacity at low light intensities, nudging a decrease in the rate of carbohydrate synthesis and resulting in a relative increase in amino acid content (Song, 2009; Zhen et al., 2010).

Studies have shown that when *Azolla* species were subjected to salt stress and heavy metal stress, their amino acid content was significantly increased, especially the content of glutamine (Dai, Xiong & Ma, 2009; Van Kempen et al., 2013). Van Kempen et al. (2013) reported that when *Azolla* species were subjected to salt stress, some amino acids content increased, especially proline and glutamine, and the study concluded that the increase in amino acid content can be used as indicators of salt stress in *Azolla* species. In our study, when the four fern species were grown in a low light environment, the amino acid content significantly increased, and there was a significant negative correlation with light transmittance. Therefore, we hypothesized that amino acids can also be used as indicators of weak light stress in fern species.

## Conclusions

The study’s measurement and analysis of total phenols, flavonoids, minerals and amino acids content in the dried leaves of these four edible fern species from Northeast China under four shading treatments showed that light and nutrients in the species are related. The leaves of four fern species can synthesize more total phenols and flavonoids as they adapt to higher-intensity light environment, but the accumulation of more mineral elements and amino acids corresponds to lower light conditions for these four edible fern species. Regardless of the transmittance of shading nets, our study found that MS had higher total phenols, AM had higher total amino acids content, OCA had higher flavonoids content and PAL had total minerals content. Based on current research results, future studies may attempt to explain the mechanism of changes in the content of minerals and amino acids of the fern species when grown in different light environments.

##  Supplemental Information

10.7717/peerj.8354/supp-1 Figure S1 The climate data of the experimental site before the experimental treatment (A) Average temperature (°C). (B) Average air humidity (%). (C) Average photosynthetically active radiation (µ mol s^−1^ m^−2^ ).

10.7717/peerj.8354/supp-2 Figure S2 The climate data of the experimental site during the experimental treatment (A) Average temperature (°C). (B) Average air humidity (%). (C) Average photosynthetically active radiation (µ mol s^−1^ m^−2^).

10.7717/peerj.8354/supp-3 Figure S3 Effect of shading on 7 essential amino acidscontent (mg 100 g^−1^ DW) in four fern species (A) Threonine content; (B) Valine content; (C) Methionine content; (D) Isoleucine content; (E) Leucine content; (F) Phenylalanine content; (G) Lysine content. MS, *M. struthiopteris*, AM, *A. multidentatum*, OCA, *O. cinnamomea* (L.) var. *asiatica*, PAL, *P. aquilinum* L. Kuhn var. *latiusculum*, 35% FS, 35% Full sunlight, 13% FS, 13% Full sunlight, 8% FS, 8% Full sunlight, 4% FS,4% Full sunlight. Different lowercase letters mean significant difference among different treatments in the same fern at *P* ≤ 0.05. Different uppercase letters mean significant difference between four fern species at *P* ≤ 0.05 (Duncan’s test). Error bars are ± SD (*n* = 3).

10.7717/peerj.8354/supp-4 Figure S4 Effect of shading on 10 non-essential amino acidscontent (mg 100 *g*^−1^ DW) in four fern species (A) Aspartic acid content; (B) Serine content; (C) Glutamic acid content; (D) Glycine content; (E) Alanine content; (F) Cysteine content; (G) Tyrosine content; (H) Histidine content; (I) Arginine content; (J) Proline content. MS, *M. struthiopteris*, AM, *A. multidentatum*, OCA, *O. cinnamomea* (L.) var. *asiatica*, PAL, *P. aquilinum* L. Kuhn var. *latiusculum*, 35% FS, 35% Full sunlight, 13% FS, 13% Full sunlight, 8% FS, 8% Full sunlight, 4% FS, 4% Full sunlight. Different lowercase letters mean significant difference among different treatments in the same fern at *P* ≤ 0.05. Different uppercase letters mean significant difference between four fern species at *P* ≤ 0.05 (Duncan’s test). Error bars are ± SD (*n* = 3).

10.7717/peerj.8354/supp-5 Figure S5 Correlation (Pearson) between total phenols,flavonoids content and H_2_O_2_ content in four fern species (A) Correlation between total phenols content and H_2_O_2_ content in four fern species. (B) Correlation between total flavonoids content and H_2_O_2_ content in four fern species.

10.7717/peerj.8354/supp-6 Table S1 Correlations (Pearson) between the measured indicators and transmittance of shading nets

10.7717/peerj.8354/supp-7 Supplemental Information 7 Raw data

## Additional Information and Declarations

### Data Availability

The following information was supplied regarding data availability: The raw measurements are available in the Supplementary Files.

## References

[ref-1] Agati G, Tattini M (2010). Multiple functional roles of flavonoids in photoprotection. New Phytologist.

[ref-2] Andrae-Marobela K, Ghislain FW, Okatch H, Majinda RRT (2013). Polyphenols: a diverse class of multi-target anti-HIV-1 agents. Current Drug Metabolism.

[ref-3] Bystriakova N, Bader M, Coomes DA (2011). Long-term tree fern dynamics linked to disturbance and shade tolerance. Journal of Vegetation Science.

[ref-4] Chen CL, Luo XH, Jin GR, Cheng Z, Pan XY, Zhu GL, Li S, Zhu YG, Tang NN (2017). Shading effect on survival, growth, and contents of secondary metabolites in micropropagated *Anoectochilus* plantlets. Brazilian Journal of Botany.

[ref-5] Chen L, Teng H, Xie ZL, Cao H, Cheang WS, Skalicka-Woniak K, Georgiev MI, Xiao JB (2018). Modifications of dietary flavonoids towards improved bioactivity: an update on structure–activity relationship. Critical Reviews in Food Science and Nutrition.

[ref-6] Colonna E, Rouphael Y, Barbieri G, De Pascale S (2016). Nutritional quality of ten leafy vegetables harvested at two light intensities. Food Chemistry.

[ref-7] Dai LP, Xiong ZT, Ma HH (2009). Effects of cadmium on nitrogen metabolism in *Azolla imbricate-Anabaena azollae* symbiosis. Acta Ecologica Sinica.

[ref-8] Dong R, Liu S, Wen LK, Zhao SC, Fu L (1993). Determination of nutrient elements and health value of dried *Pteridium aquilinum* and *Athyrium multidentatum*. Chinese Wild Plant Resources.

[ref-9] Fiutak G, Michalczyk M, Filipczak-Fiutak M, Fiedor L, Surowka K (2019). The impact of LED lighting on the yield, morphological structure and some bioactive components in alfalfa (*Medicago sativa* L.) sprouts. Food Chemistry.

[ref-10] Franzoni G, Trivellini A, Bulgari R, Cocetta G, Ferrante A, Khan MIR, Reddy PS, Ferrante A, Khan NA (2019). Chapter 10—bioactive molecules as regulatory signals in plant responses to abiotic stresses. Plant signaling molecules.

[ref-11] Frede K, Schreiner M, Baldermann S (2019). Light quality-induced changes of carotenoid composition in pak choi Brassica rapa ssp. chinensis. Journal of Photochemistry and Photobiology B—Biology.

[ref-12] Fukuda N, Anpo M, Fukuda H, Wada T (2019). Chapter 2.2—plant growth and physiological responses to light conditions. Plant factory using artificial light.

[ref-13] Galieni A, Di Mattia C, De Gregorio M, Speca S, Mastrocola D, Pisante M, Stagnari F (2015). Effects of nutrient deficiency and abiotic environmental stresses on yield, phenolic compounds and antiradical activity in lettuce (*Lactuca sativa* L.). Scientia Horticulturae.

[ref-14] Gao S, Wang Y, Yu S, Huang Y, Liu H, Chen W, He X (2020). Effects of drought stress on growth, physiology and secondary metabolites of Two *Adonis* species in Northeast China. Scientia Horticulturae.

[ref-15] Georgieva K, Dagnon S, Gesheva E, Bojilov D, Mihailova G, Doncheva S (2017). Antioxidant defense during desiccation of the resurrection plant *Haberlea rhodopensis*. Plant Physiology and Biochemistry.

[ref-16] Graham TL (1998). Flavonoid and flavonol glycoside metabolism in *Arabidopsis*. Plant Physiology and Biochemistry.

[ref-17] Han XZ, Ma R, Chen Q, Jin X, Jin YZ, An RB, Piao XM, Lian ML, Quan LH, Jiang J (2018). Anti-inflammatory action of *Athyrium multidentatum* extract suppresses the LPS-induced TLR4 signaling pathway. Journal of Ethnopharmacology.

[ref-18] Hänsch R, Mendel RR (2009). Physiological functions of mineral micronutrients (Cu, Zn, Mn, Fe, Ni, Mo, B, Cl). Current Opinion in Plant Biology.

[ref-19] Heim KE, Tagliaferro AR, Bobilya DJ (2002). Flavonoid antioxidants: chemistry, metabolism and structure–activity relationships. The Journal of Nutritional Biochemistry.

[ref-20] Hildebrandt Tatjana M, Nunes Nesi A, Araújo Wagner L, Braun H-P (2015). Amino acid catabolism in plants. Molecular Plant.

[ref-21] Jones C, Hartley S (1999). A protein competition model of phenolic allocation. Oikos.

[ref-22] Jones J, Wolf B, Mills HA (1996). Plant analysis handbook. A practical sampling, preparation, analysis, and interpretation guide.

[ref-23] Kaiser E, Weerheim K, Schipper R, Dieleman JA (2019). Partial replacement of red and blue by green light increases biomass and yield in tomato. Scientia Horticulturae.

[ref-24] Kataria S, Baghel L, Jain M, Guruprasad KN (2019). Magnetopriming regulates antioxidant defense system in soybean against salt stress. Biocatalysis and Agricultural Biotechnology.

[ref-25] Kumari R, Singh S, Agrawal SB (2009). Combined effects of Psoralens and ultraviolet-B on growth, pigmentation and biochemical parameters of *Abelmoschus esculentus* L. Ecotoxicology and Environmental Safety.

[ref-26] Kyriacou MC, Rouphael Y, Di Gioia F, Kyratzis A, Serio F, Renna M, De Pascale S, Santamaria P (2016). Micro-scale vegetable production and the rise of microgreens. Trends in Food Science & Technology.

[ref-27] Lefsrud MG, Kopsell DA, Kopsell DE, Curran-Celentano J (2006). Irradiance levels affect growth parameters and carotenoid pigments in kale and spinach grown in a controlled enviroment. Physiologia Plantarum.

[ref-28] Liao X, Greenspan P, Pegg RB (2019). Characterizing the phenolic constituents and antioxidant capacity of *Georgia peaches*. Food Chemistry.

[ref-29] Liu B, Li X (1995). Resources of economic plant *Pteridophyte* in Northeast China. Chinese Wild Plant Resources.

[ref-30] Liu HX, Yu P, Wang HZ (2011). Determination of amino acid content in five species of fern by HPLC with precolumn derivation. Amino Acids & Biotic Resources.

[ref-31] Liu Y, Fang S, Yang W, Shang X, Fu X (2018). Light quality affects flavonoid production and related gene expression in *Cyclocarya paliurus*. Journal of Photochemistry and Photobiology B: Biology.

[ref-32] Liu Y, Wang D (2018). Investigation on survival environment of *Osmunda cinnamomea* in high cold forest area. Forestry Science & Technology.

[ref-33] Maathuis FJM (2009). Physiological functions of mineral macronutrients. Current Opinion in Plant Biology.

[ref-34] Manoj GS, Murugan K (2012). Phenolic profiles, antimicrobial and antioxidant potentiality of methanolic extract of a liverwort, *Plagiochila beddomei* Steph. Indian Journal of Natural Products and Resources.

[ref-35] Meini M-R, Cabezudo I, Boschetti CE, Romanini D (2019). Recovery of phenolic antioxidants from Syrah grape pomace through the optimization of an enzymatic extraction process. Food Chemistry.

[ref-36] Mir-Marqués A, Cervera ML, De la Guardia M (2016). Mineral analysis of human diets by spectrometry methods. TrAC Trends in Analytical Chemistry.

[ref-37] Mullineaux PM, Exposito-Rodriguez M, Laissue PP, Smirnoff N (2018). ROS-dependent signalling pathways in plants and algae exposed to high light: comparisons with other eukaryotes. Free Radical Biology and Medicine.

[ref-38] Naikoo MI, Dar MI, Raghib F, Jaleel H, Ahmad B, Raina A, Khan FA, Naushin F, Khan MIR, Reddy PS, Ferrante A, Khan NA (2019). Chapter 9—role and regulation of plants phenolics in abiotic stress tolerance: an overview. Plant signaling molecules.

[ref-39] Otálora G, Piñero MC, López-Marín J, Varó P, Del Amor FM (2018). Effects of foliar nitrogen fertilization on the phenolic, mineral, and amino acid composition of escarole (*Cichorium endivia* L. var. *latifolium*). Scientia Horticulturae.

[ref-40] Paiva CL, Queiroz VAV, Simeone MLF, Schaffert RE, De Oliveira AC, Da Silva CS (2017a). Mineral content of sorghum genotypes and the influence of water stress. Food Chemistry.

[ref-41] Paiva CL, Vieira Queiroz VA, Ferreira Simeone ML, Schaffert RE, De Oliveira AC, Da Silva CS (2017b). Mineral content of sorghum genotypes and the influence of water stress. Food Chemistry.

[ref-42] Perez-Lopez U, Sgherri C, Miranda-Apodaca J, Micaelli F, Lacuesta M, Mena-Petite A, Quartacci MF, Munoz-Rueda A (2018). Concentration of phenolic compounds is increased in lettuce grown under high light intensity and elevated CO2. Plant Physiology and Biochemistry.

[ref-43] Pinto-Marijuan M, Munne-Bosch S (2014). Photo-oxidative stress markers as a measure of abiotic stress-induced leaf senescence: advantages and limitations. Journal of Experimental Botany.

[ref-44] Pourcel L, Routaboul J-M, Cheynier V, Lepiniec L, Debeaujon I (2007). Flavonoid oxidation in plants: from biochemical properties to physiological functions. Trends in Plant Science.

[ref-45] Prado C, Ponce SC, Pagano E, Prado FE, Rosa M (2016). Differential physiological responses of two *Salvinia* species to hexavalent chromium at a glance. Aquatic Toxicology.

[ref-46] Prado C, Rosa M, Pagano E, Prado F (2013). Metabolic interconnectivity among alternative respiration, residual respiration, carbohydrates and phenolics in leaves of *Salvinia minima* exposed to Cr(VI). Environmental and Experimental Botany.

[ref-47] Qi GY, Yang LQ, Xiao CX, Shi J, Mi YS, Liu XB (2015). Nutrient values and bioactivities of the extracts from three fern species in China: a comparative assessment. Food & Function.

[ref-48] Riachi LG, Simas DLR, Coelho GC, Marcellini PS, Ribeiro da Silva AJ, Bastos de Maria CA (2018). Effect of light intensity and processing conditions on bioactive compounds in maté extracted from yerba mate (Ilex paraguariensis A. St.-Hil.). Food Chemistry.

[ref-49] Riga P, Benedicto L, Gil-Izquierdo A, Collado-Gonzalez J, Ferreres F, Medina S (2019). Diffuse light affects the contents of vitamin C, phenolic compounds and free amino acids in lettuce plants. Food Chemistry.

[ref-50] Rouphael Y, Kyriacou MC, Petropoulos SA, Pascale SDe, Colla G (2018). Improving vegetable quality in controlled environments. Scientia Horticulturae.

[ref-51] Ruangrak E, Khummueng W (2019). Effects of artificial light sources on accumulation of phytochemical contents in hydroponic lettuce. Journal of Horticultural Science & Biotechnology.

[ref-52] Saito K, Yonekura-Sakakibara K, Nakabayashi R, Higashi Y, Yamazaki M, Tohge T, Fernie AR (2013). The flavonoid biosynthetic pathway in *Arabidopsis*: structural and genetic diversity. Plant Physiology and Biochemistry.

[ref-53] Sarker U, Oba S (2018). Response of nutrients, minerals, antioxidant leaf pigments, vitamins, polyphenol, flavonoid and antioxidant activity in selected vegetable amaranth under four soil water content. Food Chemistry.

[ref-54] Schenke D, Utami HP, Zhou Z, Gallegos M-T, Cai D (2019). Suppression of UV-B stress induced flavonoids by biotic stress: is there reciprocal crosstalk?. Plant Physiology and Biochemistry.

[ref-55] Shao L (2010). Effect of antioxidative materials in leaves of different *Amaranth* cultivars (*Amaranthus tricolor* L.) under low light. Journal of Zhaoqing University.

[ref-56] Shao QS, Wang HZ, Guo HP, Zhou AC, Huang YQ, Sun YL, Li MY (2014). Effects of shade treatments on photosynthetic characteristics, chloroplast ultrastructure, and physiology of *Anoectochilus roxburghii*. PLOS ONE.

[ref-57] Shibuya T, Kishigami S, Endo R, Matsuda R (2019). Interaction between red to far-red ratio of light and vapor-pressure deficit on extension growth of cucumber seedlings. Scientia Horticulturae.

[ref-58] Siracusa L, Ruberto G, Watson RR (2014). Chapter 2—plant polyphenol profiles as a tool for traceability and valuable support to biodiversity. Polyphenols in plants.

[ref-59] Soares C, Carvalho MEA, Azevedo RA, Fidalgo F (2018). Plants facing oxidative challenges—a little help from the antioxidant networks. Environmental and Experimental Botany.

[ref-60] Sonawala U, Dinkeloo K, Danna CH, McDowell JM, Pilot G (2018). Review: functional linkages between amino acid transporters and plant responses to pathogens. Plant Science.

[ref-61] Song LB, Ma QP, Zou ZW, Sun K, Yao YT, Tao JH, Kaleri NA, Li XH (2017). Molecular link between leaf coloration and gene expression of flavonoid and carotenoid biosynthesis in *Camellia sinensis* cultivar ‘Huangjinya’. Frontiers in Plant Science.

[ref-62] Song YX (2009). Effect of shading and restoration light intensity on photosynthetic characteristics, nitrogen metabolism, yield and quality in various relay cropping soybean Master.

[ref-63] Stagnari F, Galieni A, Cafiero G, Pisante M (2014). Application of photo-selective films to manipulate wavelength of transmitted radiation and photosynthate composition in red beet (*Beta vulgaris* var. *conditiva* Alef.). Journal of the Science of Food and Agriculture.

[ref-64] Stagnari F, Galieni A, Pisante M (2015). Shading and nitrogen management affect quality, safety and yield of greenhouse-grown leaf lettuce. Scientia Horticulturae.

[ref-65] Szymańska R, Ślesak I, Orzechowska A, Kruk J (2017). Physiological and biochemical responses to high light and temperature stress in plants. Environmental and Experimental Botany.

[ref-66] Tounekti T, Khemira H (2015). NaCl stress-induced changes in the essential oil quality and abietane diterpene yield and composition in common sage. Journal of Intercultural Ethnopharmacology.

[ref-67] Tounekti T, Munne-Bosch S, Vadel AM, Chtara C, Khemira H (2010). Influence of ionic interactions on essential oil and phenolic diterpene composition of Dalmatian sage (*Salvia officinalis* L.). Plant Physiology and Biochemistry.

[ref-68] Treutter D (2006). Significance of flavonoids in plant resistance: a review. Environmental Chemistry Letters.

[ref-69] Vaknin H, Bar-Akiva A, Ovadia R, Nissim-Levi A, Forer I, Weiss D, Oren-Shamir M (2005). Active anthocyanin degradation in *Brunfelsia calycina* (yesterday–today–tomorrow) flowers. Planta.

[ref-70] Van Kempen MML, Smolders AJP, Bogemann GM, Lamers LLM, Visser EJW, Roelofs JGM (2013). Responses of the *Azolla filiculoides* Stras.-*Anabaena azollae* Lam., association to elevated sodium chloride concentrations: amino acids as indicators for salt stress and tipping point. Aquatic Botany.

[ref-71] Wahle KWJ, Brown I, Rotondo D, Heys SD, Giardi MT, Rea G, Berra B (2010). Plant phenolics in the prevention and treatment of cancer. Bio-farms for nutraceuticals: functional food and safety control by biosensors.

[ref-72] Wang H, Wu S (2013). Preparation and antioxidant activity of *Pteridium aquilinum*-derived oligosaccharide. International Journal of Biological Macromolecules.

[ref-73] Wang YL, Gao SS, He XY, Li Y, Li PY, Zhang Y, Chen W (2019). Growth, secondary metabolites and enzyme activity responses of two edible fern species to drought stress and rehydration in Northeast China. Agronomy-Basel.

[ref-74] Williams RJ, Spencer JPE, Rice-Evans C (2004). Flavonoids: antioxidants or signalling molecules?. Free Radical Biology and Medicine.

[ref-75] Xiang J, Apea-Bah FB, Ndolo VU, Katundu MC, Beta T (2019). Profile of phenolic compounds and antioxidant activity of finger millet varieties. Food Chemistry.

[ref-76] Xiao J (2017). Dietary flavonoid aglycones and their glycosides: which show better biological significance?. Critical Reviews in Food Science and Nutrition.

[ref-77] Yang JH, Li ZY, Wang L, Zhang L (2002). Compositions of amino acids in two kinds of lobelia plant from Yunnan. Journal of Yunnan Minzu University (Natural Sciences Edition).

[ref-78] Yao YX (2003). Analysis of nutritive components of *Kochia scopania* (L.) and *Pteridium aquilinum* (L.). Amino Acids & Biotic Resources.

[ref-79] Yao YX, Cai JP, Li ZM, Wang YH (2003). Analysis of the nutritional components of four edible wild vegetables. Acta Nutrimenta Sinica.

[ref-80] Zhang M, Cao J, Dai X, Chen X, Wang Q (2012). Flavonoid contents and free radical scavenging activity of extracts from leaves, stems, rachis and roots of *Dryopteris erythrosora*. Iranian Journal of Pharmaceutical Research.

[ref-81] Zhao ML, Liu MC, Zhong QW, He HJ, Ji YM, Li L (2018). 29 *Jerusalem artichoke* germplasm resources amino acid content and nutritional value evaluation. Seed.

[ref-82] Zhao SC, Fu L, Liu ML (1991). Analysis of amino acids and inorganic elements in *Pteridium aquilinum*. Food Science.

[ref-83] Zhen YD, Yu M, Xiao HD, Wang HZ, Xie HS, Zeng XF (2010). Influences of silicon on content of soluble sugars, amino acids in Turf-grasses under shading stress. Journal of Huazhong Agricultural University.

[ref-84] Zhu LJ, Yan F, Chen JP, Zhang N, Zhang X, Yao XS (2016). 8-O-4’ Neolignan glycosides from the aerial parts of *Matteuccia struthiopteris*. Chinese Chemical Letters.

[ref-85] Zrig A, Tounekti T, AbdElgawad H, Hegab MM, Ali SO, Khemira H (2016). Essential oils, amino acids and polyphenols changes in salt-stressed *Thymus vulgaris* exposed to open-field and shade enclosure. Industrial Crops and Products.

