# Supplementary figures and images for: Response of total phenols, flavonoids, minerals, and amino acids of four edible fern species to four shading treatments
Source: PeerJ. 2020 Jan 13;8:e8354. doi: 10.7717/peerj.8354 (PMC6964689; doi:10.7717/peerj.8354)

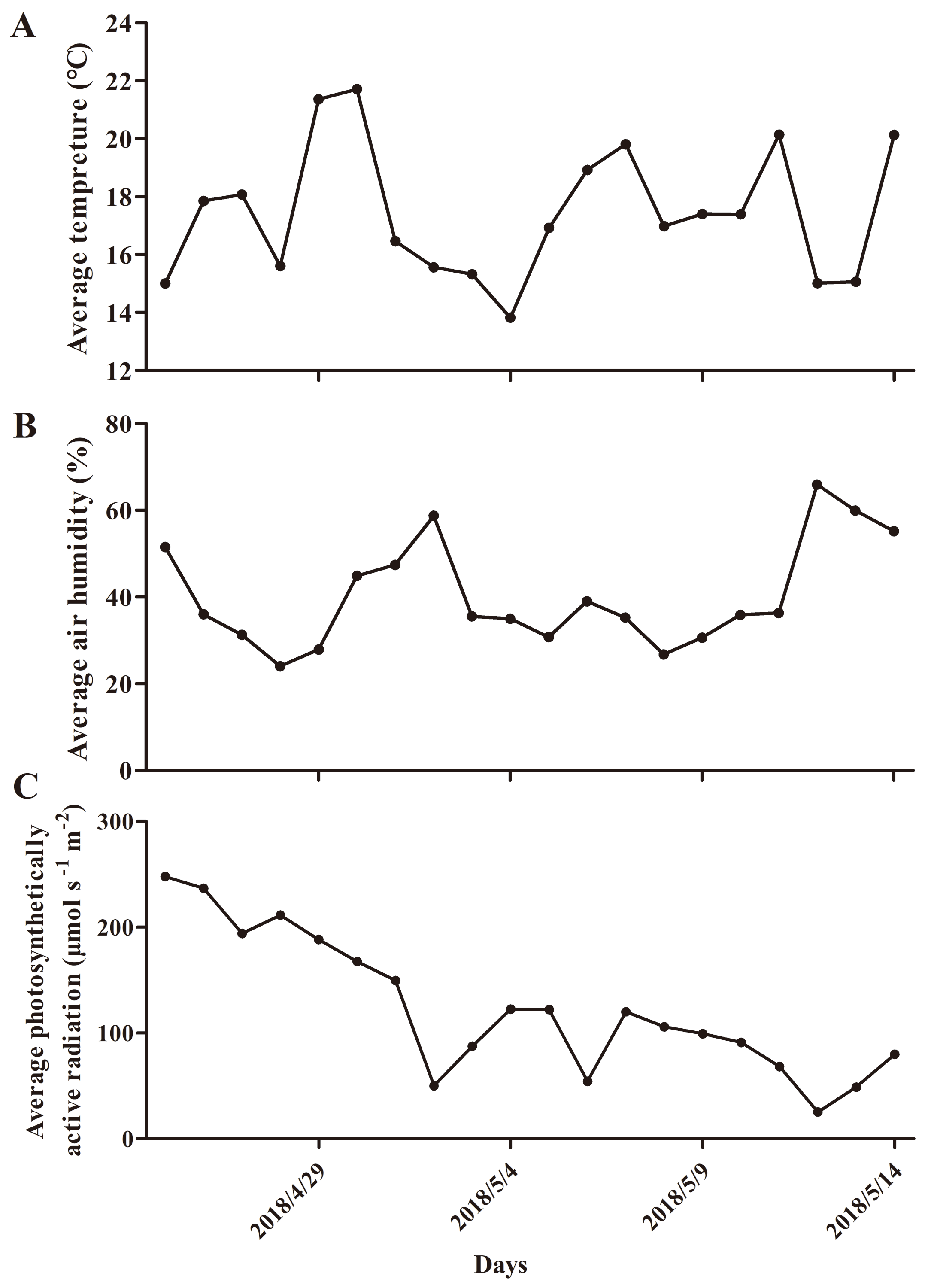

Supplement: Figure S1 — (A) Average temperature (°C). (B) Average air humidity (%). (C) Average photosynthetically active radiation (µ mol s−1 m−2 ). [file peerj-08-8354-s001.png]

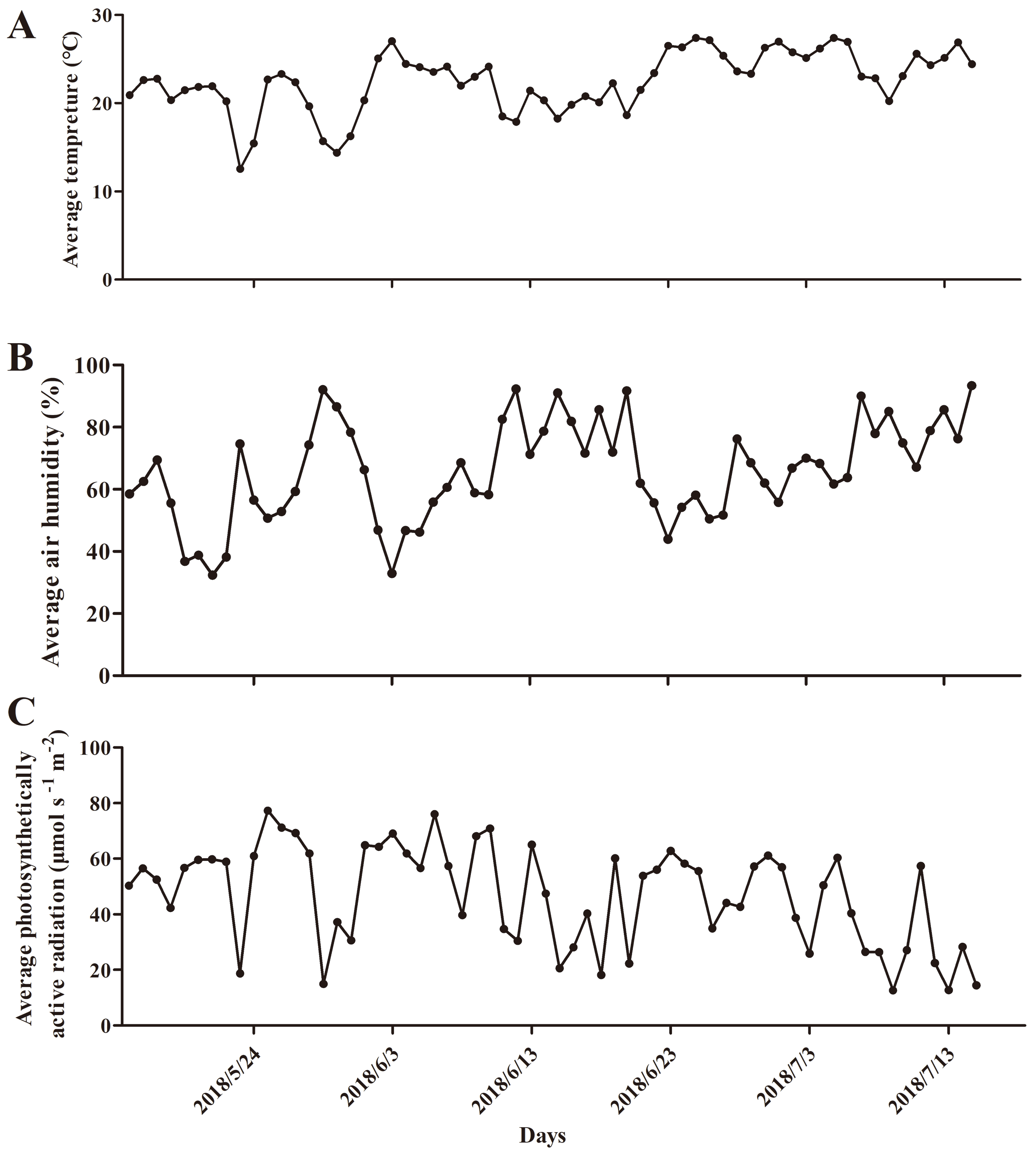

Supplement: Figure S2 — (A) Average temperature (°C). (B) Average air humidity (%). (C) Average photosynthetically active radiation (µ mol s−1 m−2). [file peerj-08-8354-s002.png]

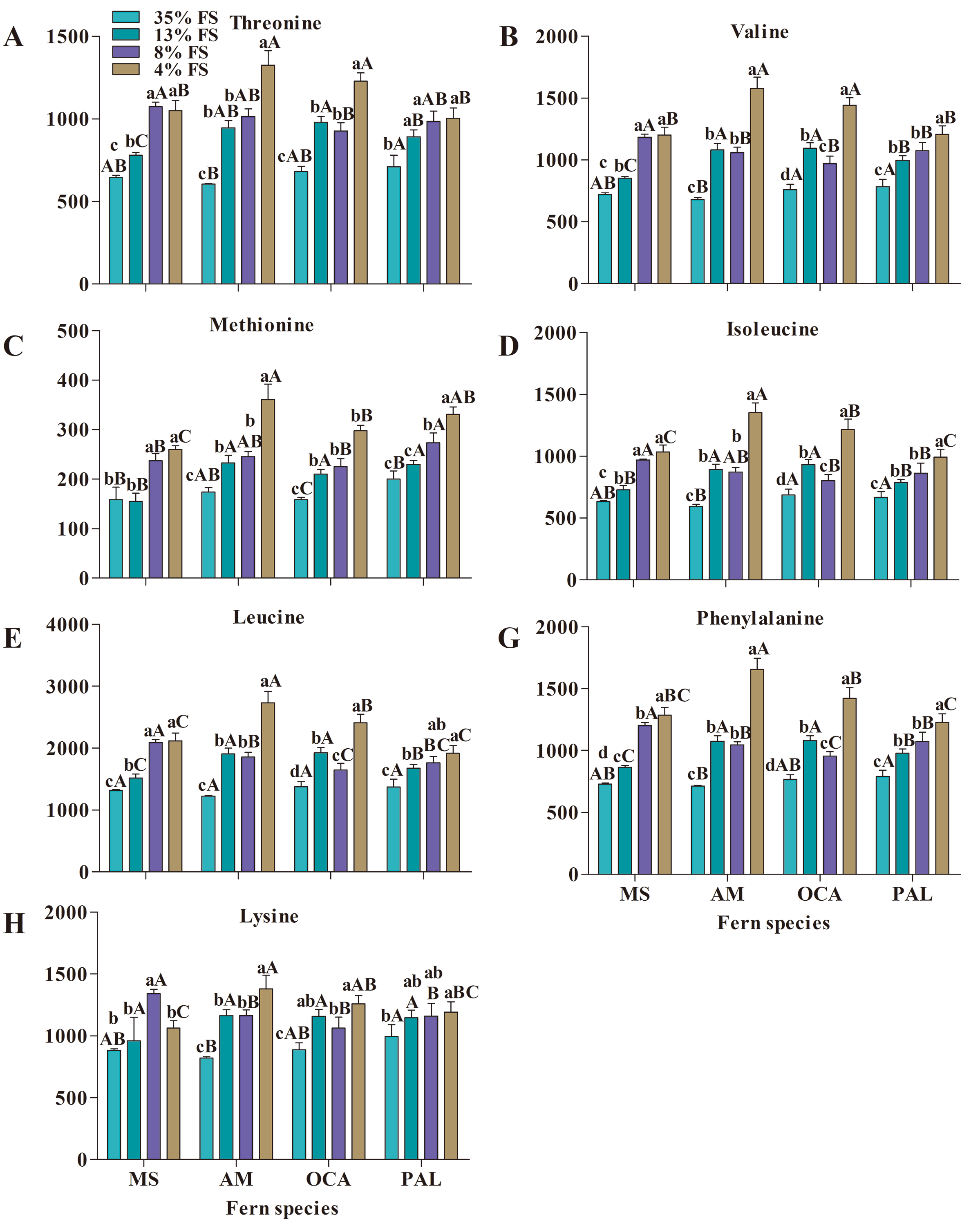

Supplement: Figure S3 — (A) Threonine content; (B) Valine content; (C) Methionine content; (D) Isoleucine content; (E) Leucine content; (F) Phenylalanine content; (G) Lysine content. MS, M. struthiopteris, AM, A. multidentatum, OCA, O. cinnamomea (L.) var. asiatica, PAL, P. aquilinum L. Kuhn var. latiusculum, 35% FS, 35% Full sunlight, 13% FS, 13% Full sunlight, 8% FS, 8% Full sunlight, 4% FS,4% Full sunlight. Different lowercase letters mean significant difference among different treatments in the same fern at P ≤ 0.05. Different uppercase letters mean significant difference between four fern species at P ≤ 0.05 (Duncan’s test). Error bars are ± SD (n = 3). [file peerj-08-8354-s003.png]

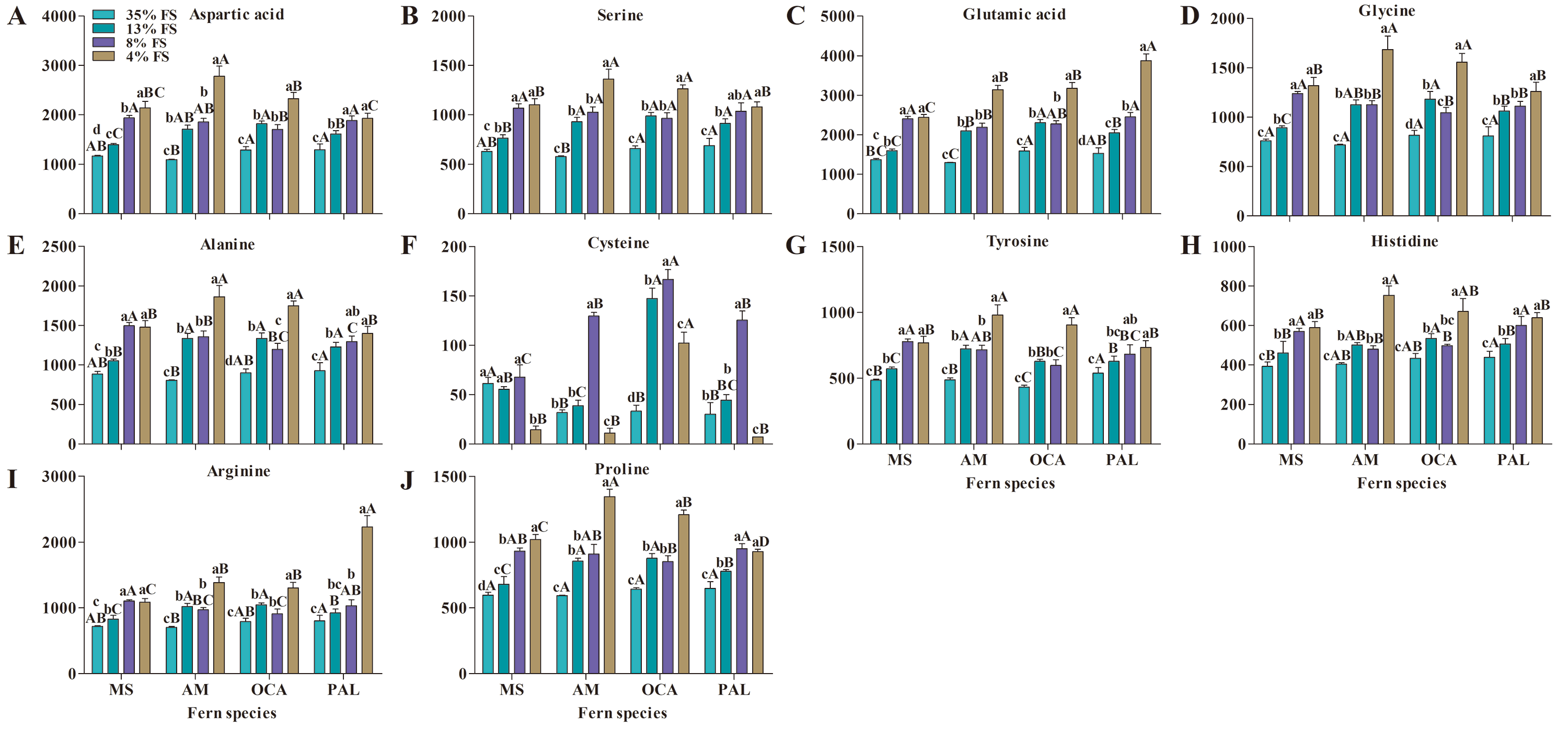

Supplement: Figure S4 — (A) Aspartic acid content; (B) Serine content; (C) Glutamic acid content; (D) Glycine content; (E) Alanine content; (F) Cysteine content; (G) Tyrosine content; (H) Histidine content; (I) Arginine content; (J) Proline content. MS, M. struthiopteris, AM, A. multidentatum, OCA, O. cinnamomea (L.) var. asiatica, PAL, P. aquilinum L. Kuhn var. latiusculum, 35% FS, 35% Full sunlight, 13% FS, 13% Full sunlight, 8% FS, 8% Full sunlight, 4% FS, 4% Full sunlight. Different lowercase letters mean significant difference among different treatments in the same fern at P ≤ 0.05. Different uppercase letters mean significant difference between four fern species at P ≤ 0.05 (Duncan’s test). Error bars are ± SD (n = 3). [file peerj-08-8354-s004.png]

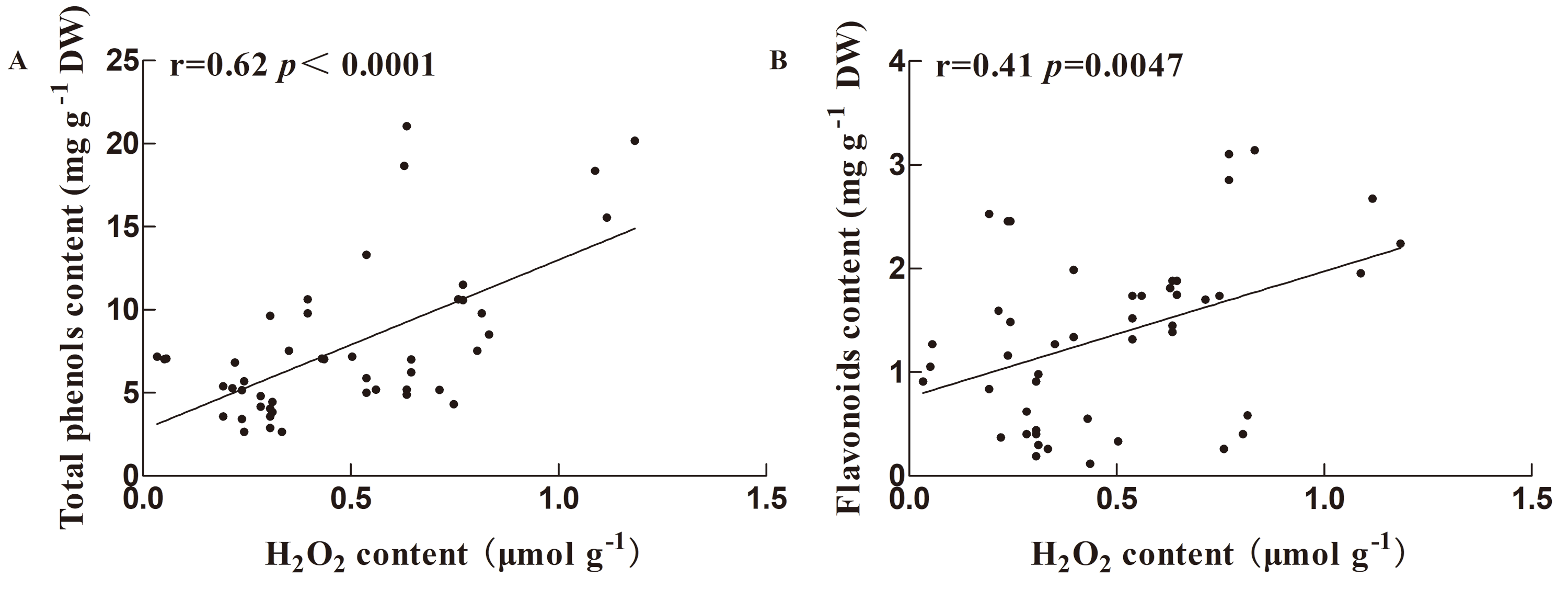

Supplement: Figure S5 — (A) Correlation between total phenols content and H2O2 content in four fern species. (B) Correlation between total flavonoids content and H2O2 content in four fern species. [file peerj-08-8354-s005.png]
