# Supplementary material for: Response of total phenols, flavonoids, minerals, and amino acids of four edible fern species to four shading treatments
Source: PeerJ. 2020 Jan 13;8:e8354. doi: 10.7717/peerj.8354 (PMC6964689; doi:10.7717/peerj.8354)
Supplement: Table S1 [file peerj-08-8354-s006.docx]

Supplemental Table S1 **Correlations (Pearson) between the measured indicators and transmittance of shading nets.**

| Indicators | Correlation coefficient | *p* value |
| --- | --- | --- |
| Total phenols content (mg g^-1^ DW) | 0.69 | ＜0.0001 |
| Flavonoids content (mg g^-1^ DW) | 0.52 | 0.0002 |
| K content (mg g^-1^ DW) | -0.45 | 0.0012 |
| Ca content (mg g^-1^ DW) | -0.08 | 0.5660 |
| Mg content (mg g^-1^ DW) | -0.04 | 0.7738 |
| Fe content (mg g^-1^ DW) | -0.68 | ＜0.0001 |
| Mn content (mg g^-1^ DW) | -0.15 | 0.2879 |
| Cu content (mg g^-1^ DW) | -0.70 | ＜0.0001 |
| Zn content (mg g^-1^ DW) | -0.58 | ＜0.0001 |
| Na content (mg g^-1^ DW) | -0.36 | 0.0125 |
| Total minerals content (mg g^-1^ DW) | -0.55 | ＜0.0001 |
| Threonine content (mg 100g^-1^ DW) | -0.86 | ＜0.0001 |
| Valine content (mg 100g^-1^ DW) | -0.82 | ＜0.0001 |
| Methionine content (mg 100g^-1^ DW) | -0.73 | ＜0.0001 |
| Isoleucine content (mg 100g^-1^) | -0.77 | ＜0.0001 |
| Leucine (mg 100g^-1^ DW) | -0.78 | ＜0.0001 |
| Phenylalanine (mg 100g^-1^ DW) | -0.80 | ＜0.0001 |
| Lysine(mg 100g^-1^ DW) | -0.74 | ＜0.0001 |
| Aspartic acid (mg 100g^-1^ DW) | -0.82 | ＜0.0001 |
| Serine (mg 100g^-1^ DW) | -0.88 | ＜0.0001 |
| Glutamic acid (mg 100g^-1^ DW) | -0.79 | ＜0.0001 |
| Glycine (mg 100g^-1^ DW) | -0.81 | ＜0.0001 |
| Alanine (mg 100g^-1^ DW) | -0.84 | ＜0.0001 |
| Cysteine (mg 100g^-1^ DW) | -0.23 | 0.1162 |
| Tyrosine (mg 100g^-1^ DW) | -0.80 | ＜0.0001 |
| Histidine (mg 100g^-1^ DW) | -0.76 | ＜0.0001 |
| Arginine (mg 100g^-1^ DW) | -0.60 | ＜0.0001 |
| Proline (mg 100g^-1^ DW) | -0.80 | ＜0.0001 |
| Total essential amino acids content (mg 100g^-1^ DW) | -0.84 | ＜0.0001 |
| Total non-essential amino acids content  (mg 100g^-1^ DW) | -0.81 | ＜0.0001 |
| Total amino acids content (mg 100g^-1^ DW) | -0.84 | ＜0.0001 |
